# Vasopressin receptors V1_a_ and V2 are not osmosensors

**DOI:** 10.14814/phy2.12519

**Published:** 2015-08-26

**Authors:** Kasper Lykke, Mette Assentoft, Robert A Fenton, Mette M Rosenkilde, Nanna MacAulay

**Affiliations:** 1 Department of Cellular and Molecular Medicine, Faculty of Medical and Health Sciences, University of CopenhagenCopenhagen, Denmark; 2 Department of Biomedicine and InterPrET Center, Aarhus UniversityAarhus, Denmark; 3 Department of Neuroscience and Pharmacology, Faculty of Medical and Health Sciences, University of CopenhagenCopenhagen, Denmark

**Keywords:** G protein-coupled receptors, osmosensing, vasopressin, volume regulation

## Abstract

Herein, we investigated whether G protein-coupled signaling via the vasopressin receptors of the V1_a_ and V2 subtypes (V1_a_R and V2R) could be obtained as a direct response to hyperosmolar challenges and/or whether hyperosmolar challenges could augment classical vasopressin-dependent V1_a_R signaling. The V1_a_R-dependent response was monitored indirectly via its effects on aquaporin 4 (AQP4) when heterologously expressed in *Xenopus* oocytes and V1_a_R and V2R function was directly monitored following heterologous expression in COS-7 cells. A tendency toward an osmotically induced, V1_a_R-mediated reduction in AQP4-dependent water permeability was observed, although osmotic challenges failed to mimic vasopressin-dependent V1_a_R-mediated internalization of AQP4. Direct monitoring of inositol phosphate (IP) production of V1_a_R-expressing COS-7 cells demonstrated an efficient vasopressin-dependent response that was, however, independent of hyperosmotic challenges. Similarly, the cAMP production by the V2R was unaffected by hyperosmotic challenges although, in contrast to the V1_a_R, the V2R displayed an ability to support alternative signaling (IP production) at higher concentration of vasopressin. V1_a_R and V2R respond directly to vasopressin exposure, but they do not have an ability to act as osmo- or volume sensors when exposed to an osmotic gradient in the absence or presence of vasopressin.

## Introduction

The antidiuretic peptide hormone arginine vasopressin participates in the regulation of mammalian water homeostasis. It is released systemically and centrally (via vasopressinergic neurons located in direct contact with the blood vessels; Jojart et al. 1984) as a regulatory response to increases in plasma osmolarity or a reduction in blood volume (Landgraf 1992). Vasopressin exerts its effects via a family of G protein-coupled receptors. The most prominently expressed are the V1_a_ and the V2 type (V1_a_R and V2R) (Lolait et al. 1995). The V1_a_R is found in a number of tissues including brain; it has been detected in neurons, glial cells, and endothelial cells of the blood–brain barrier (van Leeuwen et al. 1987; Chen et al. 1993; Szmydynger-Chodobska et al. 2004). The V2R has a more restricted distribution, and is predominantly expressed in the kidney (Lolait et al. 1992). Vasopressin receptors recruit different G proteins and hence confer distinct signaling pathways: activation of the V1_a_R leads to G_q_ recruitment, increased activity of phospholipase C*β* (PLC*β*), generation of inositol trisphosphate (IP_3_), Ca^2+^ release from intracellular stores, and protein kinase C (PKC) activation (Zhao and Brinton 2003). Conversely, V2R-activation leads to G_s_ recruitment, activation of adenylyl cyclase, and cAMP generation, followed by increased catalytic activity of cAMP-dependent protein kinases (for review, see Lolait et al. 1995). A prominent site of action for vasopressin is the kidney, in which activation of the V2R results in increased absorption of water, and ultimately helps maintain body water homeostasis, by modulating plasma membrane levels of the water channel AQP2 in collecting duct principal cells (Kortenoeven and Fenton 2014). Vasopressin, in addition, facilitates ion and water accumulation in the brain in a V1_a_R-dependent manner (Kleindienst et al. 2006; Liu et al. 2010). We have previously demonstrated a V1_a_R-mediated downregulation of aquaporin 4 (AQP4) membrane expression levels and proposed that this downregulation might be beneficial during periods of dehydration in an attempt to limit the loss of water from the brain (Moeller et al. 2009).

Since a prominent role of vasopressin receptors is to participate in volume regulation, it would be of physiological advantage for the receptor to sense the osmotic change directly in the affected cells – instead of after the appreciable delay with which vasopressin is released systemically and/or centrally. Such intrinsic osmo/volume sensing abilities have previously been described for other membrane transport proteins such as the K^+^-channel KCNQ1 (Grunnet et al. 2003), the osmosensing trimeric glycine betaine transporter, BetP (Krämer and Ziegler 2009), and several ion channels of the transient receptor potential family (Plant 2014). It has thus been proposed that vasopressin-induced activation of the V1_a_R could be enhanced by hyperosmolar conditions (Izumi et al. 2008). Here, we investigated whether V1_a_R (or V2R)-dependent signaling could be obtained as a direct response to hyperosmolar challenges and whether hyperosmolar challenges could augment classical vasopressin-dependent V1_a_R signaling.

## Materials and Methods

### Oocyte preparation and protein expression

*Xenopus laevis* frogs were obtained from Nasco (Fort Atkinson, WI) or National Center for Scientific Research (France). Oocytes were surgically removed from anesthetized frogs and prepared as previously described (Fenton et al. 2010). The protocol complies with the European Community guidelines for the use of experimental animals and the experiments were approved by The Danish National Committee for Animal Studies. Rat AQP4.M23 (obtained from S. Nielsen, Aalborg University, Denmark) and rat mGluR1 (obtained from J. P. Pin, Montpellier University, France) were subcloned into the oocyte expression vector pXOOM and the human V1_a_R (obtained from M. J. Brownstein, NIMH, Bethesda, MD) was subcloned into the vector pNB1. The cDNAs were linearized downstream from the poly-A segment and in vitro transcribed using T7 mMessage Machine (Ambion, Austin, TX). The cRNA was then extracted with MEGAclear (Ambion, Austin, TX) and microinjected into defolliculated *Xenopus* oocytes (8 ng rAQP4 RNA/oocyte, 16 ng hV1_a_R RNA/oocyte, or 16 ng rmGluR1a RNA/oocyte). The oocytes were kept in Kulori medium (in mmol/L: 90 NaCl, 1 KCl, 1 CaCl_2_, 1 MgCl_2_, 5 HEPES, pH 7.4) for 4–6 days at 19°C prior to experiments.

### Volume measurements

The experimental setup for measuring water permeability of oocytes has been described in detail previously (Zeuthen et al. 2006). Briefly, the oocyte was placed in a small chamber with a glass bottom and perfused with a control solution (in mmol/L: 100 NaCl, 2 KCl, 1 CaCl_2_, 1 MgCl_2_, 10 HEPES, pH 7.4) at room temperature. Images of the oocytes were captured continuously from below at a rate of 25 images/sec. The oocytes were challenged with a hyperosmolar solution (control solution containing an additional 50 mOsm mannitol) in order to determine the osmotic water permeability:

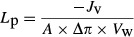


where *J*_V_ is the initial water flux during osmotic challenge, *A* is membrane surface area (nine times the apparent surface area due to membrane folding; Zampighi et al. 1995), Δ*π* is the osmotic challenge, and *V*_W_ is the partial molal volume of water (18 cm^3^/mol). All osmolarities were validated using a cryoscopic osmometer Type 15 (Löser, Berlin, Germany).

### Immunocytochemistry

Oocytes (3–6 per condition per experiment) were fixed for 1 h in 3% paraformaldehyde in Kulori medium, rinsed in Kulori medium, dehydrated in a series of ethanol concentrations (30 min in 70%, 96%, and 99%) followed by incubation in xylene for 1 h. Oocytes were infiltrated with paraffin for 1 h at 50°C before embedding. Sections of 2 *μ*m were cut on a Leica RM 2126 microtome and immunostaining performed as described previously (Fenton et al. 2010) using a rabbit polyclonal anti-AQP4 antibody 1:5000 (Alamone Laboratories, Jerusalem, Israel). An Alexa 488-conjugated secondary antibody 1:1,000 was used for visualization (DAR; Invitrogen, Nærum, Denmark). A Leica TCS SL confocal microscope and Leica confocal software were used for imaging of the oocytes. Control AQP4-expressing oocytes were used to set laser intensity and capture settings on the microscope such that saturation of images for each condition was avoided. The microscope and laser settings were kept constant for imaging of each oocyte within each experiment. Images were taken using an HCX PL APO ×63 oil objective lens (numerical aperture: 1.40). A minimum of two images per oocyte, with 3–6 oocytes per experiment, were used for statistical analysis. Image semiquantification and validation was performed as previously described (Fenton et al. 2010).

### Cell culture and transfection

COS-7 cells were grown in Dulbecco’s modified Eagle’s medium (Invitrogen) supplemented with 10% fetal calf serum, 2 mmol/L glutamine, 180 U/mL penicillin, and 45 *μ*g/mL streptomycin (10% CO_2_, 37°C). Receptor signaling experiments were carried out using COS-7 cells transfected with the human vasopressin receptors, V1_a_R or V2R in pcDNA3 (obtained from M. J. Brownstein, NIMH, Bethesda, MD). The transfection was performed using the calcium phosphate precipitation method (Graham and Van der Eb 1973) according to Kissow et al. (2012).

### Inositol phosphate assay

Inositol phosphate accumulation was measured using a scintillation proximity assay (SPA). One day after transfection, COS-7 cells were seeded at 35,000 cells/well in 96-well plates for 24 h with 0.5 *μ*Ci of *myo*-[^3^H]inositol (NET1156, PerkinElmer, Waltham, MA) in 100 *μ*L medium per well. Cells were washed twice in Hank’s balanced salt solution (HBSS, in mmol/L: 138 NaCl, 4 NaHCO_3_, 0.33 Na_2_HPO_4_, 5 KCl, 0.4 KH_2_PO_4_, 1.3 CaCl_2_, 0.5 MgCl_2_, 0.4 MgSO_4_, and 5.5 d-glucose) prior to 15 min incubation in HBSS supplemented with 10 mmol/L LiCl at 37°C to block IP1 breakdown. Subsequently cells were treated with vasopressin (in concentrations as indicated in the text) or hyperosmolar challenges (in duplicate or triplicate determinations) for 90 min at 37°C. Cells were extracted by addition of 40 *μ*L formic acid (10 mmol/L) followed by incubation on ice for 30–120 min. Thirty-five microliter of the solution in each well was transferred to new wells of a 96-well plate to which 1 mg of yttrium silicate SPA beads (SPA-Ysi; RPNQ, PerkinElmer) was added to each well and the plate mixed for 30 min by high-speed agitation allowing the generated [^3^H]inositol phosphates to bind to the SPA beads. The beads were centrifuged (5 min, 400 g) and [^3^H]inositol phosphate binding was measured on a TopCount NXT (PerkinElmer, Waltham, MA).

### cAMP assay

The day after transfection the COS-7 cells were seeded at 20,000 cells/well in 96-well plates. 24 h later, cells were washed twice with HBS (in mmol/L: 20 HEPES, 150 NaCl, 0.75 NaH_2_PO_4_, pH 7.4) and incubated 30 min at 37°C in HBS containing 1 mmol/L isobutylmethylxanthine (IBMX) phosphodiesterase inhibitor (Sigma-Aldrich, Brondby, Denmark). Vasopressin and/or hyperosmolar treatments were then added (in duplicate or triplicate determinations) followed by 30 min incubation at 37°C. Subsequently, the medium was removed and the cells were treated with the enzyme fragment complementation-based cAMP assay according to manufacturer’s instructions (HitHunter cAMP XS+ assay, DiscoveRx, Fremont, CA). The cAMP content was measured as luminescence using an EnVision 2104 Multitable Platereader (PerkinElmer) with a cAMP standard curve for validation.

### Chemicals

[Arg^8^]-vasopressin (1 mmol/L) and l-glutamate (500 mmol/L), both Sigma-Aldrich, were dissolved in water and the stock solutions kept in aliquots at −20°C.

### Statistics

EC_50_ values were obtained from dose–response curves obtained with fitting to log(agonist) versus response with variable slope (four parameters) according to the equation *Y* = Bottom + (Top–Bottom)/(1 + 10^((LogEC_50_-X) × HillSlope)) in GraphPad Prism 6.0 software (San Diego, CA). Statistical analyses were carried out in GraphPad Prism 6.0 software as described in the text. Data were obtained from at least three different animal donors (*Xenopus laevis*) or at least three different cell transfections and are presented as means ± SEM (confidence intervals [CI] when Log scales were employed). A probability level of *P* < 0.05 was considered statistically significant.

## Results

### Putative osmosensing by V1_a_R assessed by water permeability measurements

**Figure 1:**
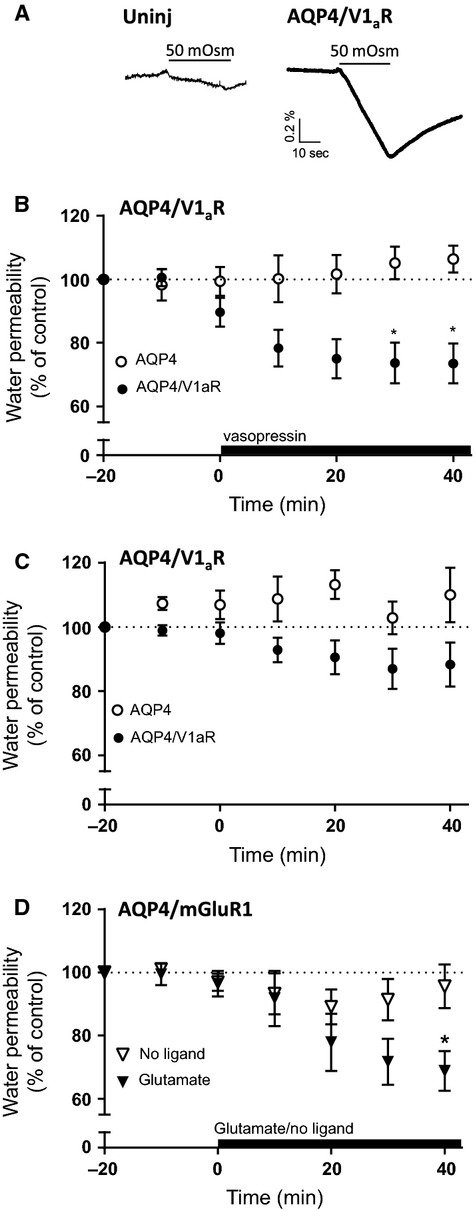
V1_a_R-dependent downregulation of AQP4. (A) Volume traces obtained from an uninjected oocyte (left panel) and an AQP4/V1_a_R-expressing oocyte challenged with an osmotic gradient of 50 mOsm mannitol for 30 sec. (B) Relative water permeability of oocytes expressing AQP4 (open circles; *n* = 5) or coexpressing AQP4/V1_a_R (filled circles, *n* = 20) exposed to 1 *μ*mol/L vasopressin as marked by the black bar. (C) Relative water permeability of oocytes expressing AQP4 (open circles; *n* = 6) or coexpressing AQP4/V1_a_R (filled circles; *n* = 22) when exposed to repeated osmotic challenges. (D) Relative water permeability of oocytes coexpressing AQP4/mGluR1a and exposed to 500 *μ*mol/L glutamate as indicated by the black bar (filled symbols, *n* = 8) or kept in control solution (open symbols, *n* = 8, not exposed to glutamate). The groups were compared with two-way analysis of variance (ANOVA) with Šídák’s multiple comparison post hoc test. *P < 0.05.

Activation of the G_q_ protein-coupled vasopressin receptor V1_a_R expressed in *Xenopus laevis* oocytes initiates a signaling cascade involving phospholipase C and increased levels of intracellular Ca^2+^ (Nathanson et al. 1992; Ancellin and Morel 1998). We have previously in the *Xenopus laevis* expression system, by several complementary technical approaches, demonstrated that activation of V1_a_R led to an internalization of coexpressed AQP4 and thus a reduction in the osmotic water permeability of the oocytes (Moeller et al. 2009). We therefore initially utilized this experimental setup to obtain a functional read-out for V1_a_R activation with the aim to determine if cell shrinkage *in itself* can mimic previously published vasopressin-dependent activation of V1_a_R in the oocytes (Moeller et al. 2009). AQP4 was expressed in the oocytes either alone (AQP4) or along with V1_a_R (AQP4/V1_a_R). The oocytes were monitored for volume changes with a sensitive camera while abruptly challenged with a hyperosmotic gradient of 50 mOsm (obtained by addition of 50 mmol/L mannitol to the test solution to increase the osmolarity while keeping the ionic strength constant). The rate of osmotically induced oocyte shrinkage increased dramatically upon expression of AQP4 (∼15-fold over that of uninjected control oocytes) and amounted to a physiologically relevant cell shrinkage of around 1% during exposure to the osmotic challenge, see representative volume traces in Figure1A. After three initial control water permeability measurements, the oocytes were exposed to saturating concentrations of vasopressin to obtain maximal activation of V1_a_R (1 *μ*mol/L, indicated by the black bar in Fig.1B) and the osmotic water permeability determined as a function of time and normalized to the control value (Fig.1B). The water permeability of AQP4/V1_a_R-expressing oocytes (*n* = 20) was reduced significantly more than those expressing only AQP4 (*n* = 5) after exposure to vasopressin for 30 min (*P* < 0.05, Fig.1B), indicating that activation of the receptor leads to downregulation of AQP4-mediated water permeability. To test if V1_a_R could directly respond to hyperosmotic challenges, AQP4/V1_a_R-expressing oocytes (*n* = 22) and AQP4-expressing oocytes (*n* = 6) were exposed to consecutive hyperosmotic challenges, as above, but in the absence of vasopressin in the test medium (Fig.1C). The osmotic water permeability of the oocytes expressing either AQP4/V1_a_R or AQP4 did not differ in their response to repeated hyperosmotic challenges, although a tendency toward a difference between the two groups was observed (*P* = 0.053). The low inherent water permeability of the uninjected oocyte was not significantly affected by exposure to vasopressin or to repeated osmotic challenges (*data not shown*). Due to the observed tendency for an osmotically induced reduction in the water permeability of AQP4/V1_a_R-expressing oocytes, a parallel control experimental series was performed with an unrelated G_q_ protein-coupled glutamate receptor (mGluR1) to determine if G_q_ protein-coupled receptors shared a general feature of hyperosmolarity-induced G_q_ protein activation. Similar to that observed with V1_a_R, the water permeability of AQP4/mGluR1-expressing oocytes was reduced upon activation of mGluR1, by addition of glutamate (500 *μ*mol/L) to the test medium, to a level significantly different from exposing the AQP4/mGluR1 to repeated hyperosmolar challenges in the absence of glutamate (*n* = 8, *P* < 0.05, Fig.1D). These data indicate that agonist-induced activation of G_q_ coupled receptors confers downregulation of AQP4-mediated water permeability which was, however, not significantly mimicked by exposure of the oocytes to repeated hyperosmolar challenges.

### Putative osmosensing by V1_a_R assessed by immunocytochemistry

**Figure 2:**
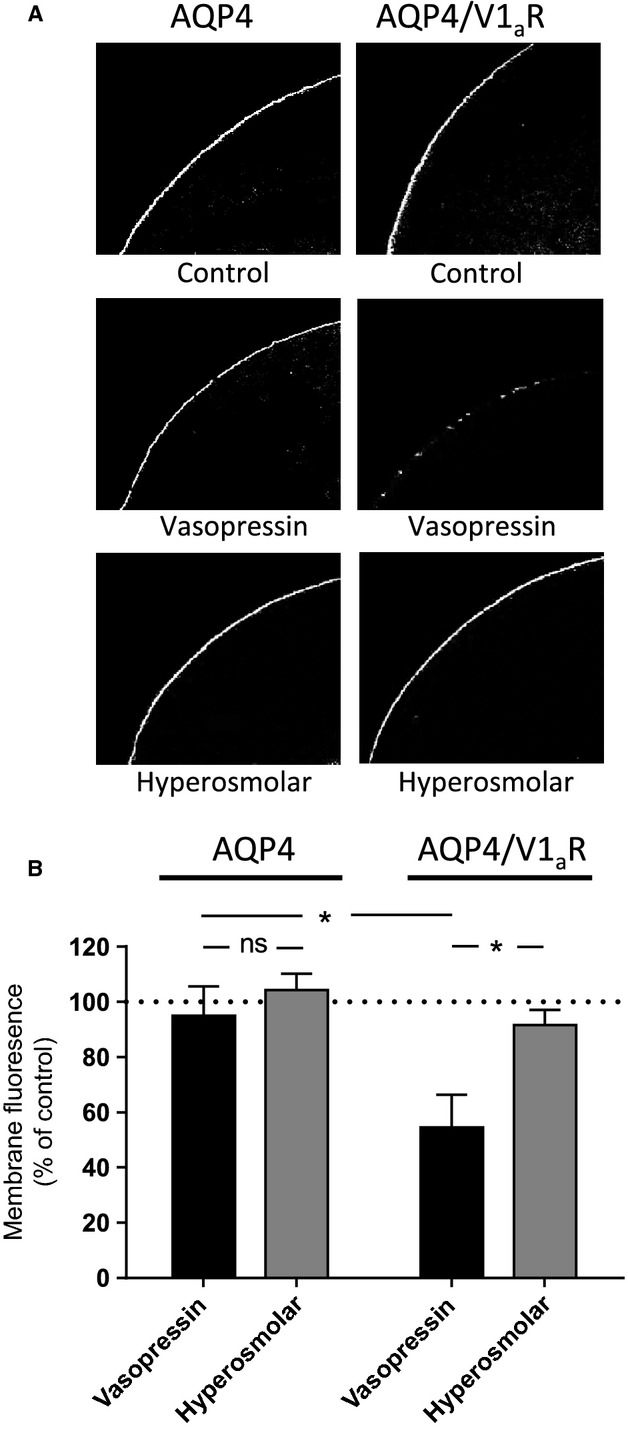
V1_a_R-dependent internalization of AQP4. (A) Confocal laser scanning microscopy of oocytes expressing either AQP4 (left panel) or AQP4/V1_a_R (right panel) immune-labeled for AQP4. The upper panels are representative images of oocytes exposed to control solution without vasopressin for 80 min. The middle panels are representative images of oocytes kept in control solution for 20 min and then treated with 1 *μ*mol/L vasopressin for 60 min. The lower panels are representative images of oocytes treated with a 50 mOsm hyperosmolar gradient for 30 sec every 10 min of an 80-min incubation period. (B) Oocyte plasma membrane fluorescence intensity normalized to that of the oocytes kept in control solution, *n* = 5 experiments with 3–6 oocytes per condition. The indicated groups were compared using one-way analysis of variance (ANOVA) with Šídák’s multiple comparison post hoc test. *P < 0.05; ns, not significant.

To assess a role of hyperosmotic challenge on the V1_a_R using an alternative approach, we analyzed the membrane abundance of AQP4 in oocytes. We have previously shown a V1_a_R-dependent internalization of AQP4 following vasopressin exposure (Moeller et al. 2009) and aimed in the present experimental series to determine if the vasopressin-dependent internalization of AQP4 could be mimicked by cell shrinkage. AQP4/V1_a_R-expressing oocytes were exposed to either vasopressin or repeated hyperosmotic challenges and the plasma membrane abundance of AQP4 subsequently quantified by immunocytochemistry. Oocytes expressing AQP4 alone or AQP4/V1_a_R were incubated for 80 min in control conditions, in the presence of 1 *μ*mol/L vasopressin for the last 60 min, or challenged with an extracellular osmotic gradient of 50 mOsm mannitol for 30 sec every 10 min during the span of the 80 min incubation time (designed to mimic the water permeability measurements from Fig.1). Representative confocal images of AQP4- and AQP4/V1_a_R-expressing oocytes are shown in Figure2A. No immune reactivity toward AQP4 was detected in uninjected oocytes (*data not shown* and Moeller et al. 2009). The plasma membrane abundance of AQP4 was quantified and normalized to the membrane abundance observed under control conditions in AQP4- and AQP4/V1_a_R-expressing oocytes, respectively (Fig.2B). Exposure to vasopressin induced a reduction in plasma membrane abundance in AQP4/V1_a_R-expressing oocytes (55 ± 12% of control, *n* = 5 experiments with 3–6 oocytes per condition) which was significantly more than that obtained in AQP4-expressing oocytes (95 ± 11% of control, *n* = 5 experiments with 3–6 oocytes per condition), *P* < 0.05. The vasopressin-induced AQP4 internalization in the AQP4/V1_a_R-expressing oocytes was not mimicked by repeated hyperosmolar challenges (compare 92 ± 5% of control for hyperosmolar challenges and the 55 ± 12% of control for vasopressin, *n* = 5 experiments with 3–6 oocytes per condition, *P* < 0.05). Oocytes expressing AQP4 alone, like-wise, did not respond to repeated hyperosmolar challenges (104 ± 6% of control, *n* = 5 experiments with 3–6 oocytes per condition). In the present experimental setting, based on an indirect experimental read-out of V1_a_R function, the V1_a_R responds to vasopressin exposure but not to hyperosmotic challenges, indicating that V1_a_R does not act as an osmosensor.

### Putative osmosensing by V1_a_R assessed by quantification of intracellular secondary messengers

**Figure 3:**
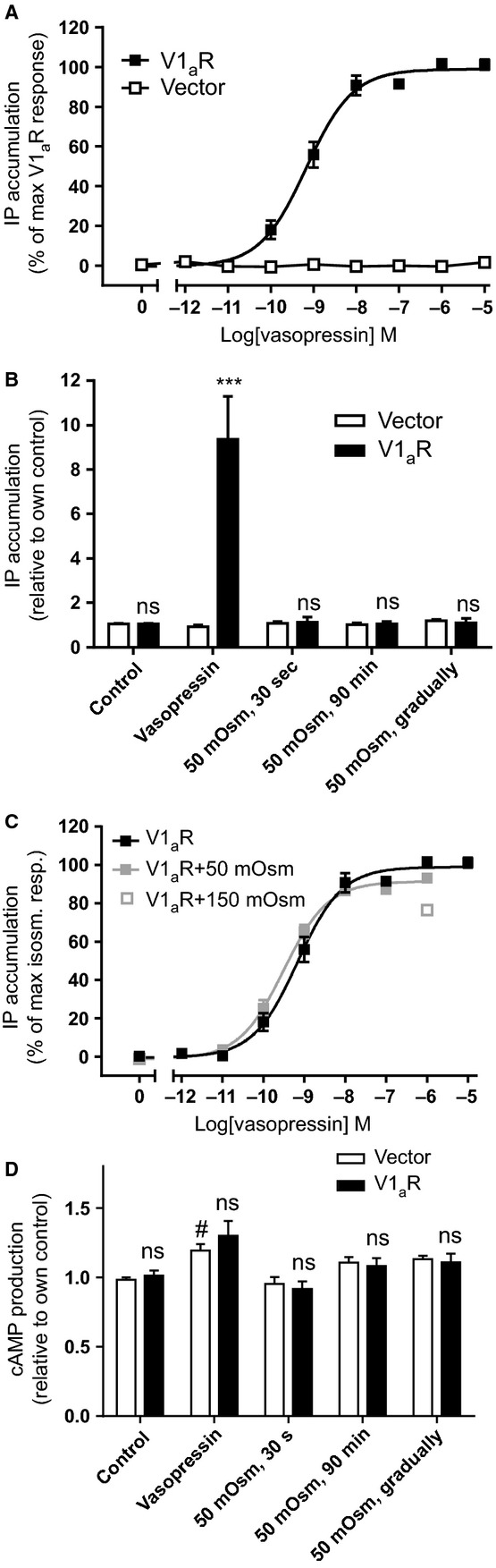
Hyperosmolar effects on V1_a_R-dependent inositol phosphate (IP) production. (A) Vasopressin dose–response curve measured with IP production in COS-7 cells transfected with either vector alone or with V1_a_R, *n* = 7. (B) IP production of COS-7 cells transfected with either vector alone or with V1_a_R upon addition of vasopressin (1 *μ*mol/L) or various hyperosmolar treatments as indicated on graph, *n* = 3–8. (C) Effect of hyperosmolar treatment (50 or 150 mOsm) on the vasopressin dose–response curve, *n* = 3–8. (D) cAMP production obtained in COS-7 cells transfected with either vector alone or with V1_a_R in response to vasopressin exposure (1 *μ*mol/L) or various hyperosmolar treatments as indicated on graph, *n* = 4–6. Data obtained with V1_a_R-transfected cells were compared to those obtained with vector-transfected cells using two-way analysis of variance (ANOVA) with Šídák’s multiple comparison post hoc test and indicated with * (note: the difference between the vasopressin-induced cAMP production in vector- and V1_a_R-transfected cells was not statistically significant even when compared with Student’s *t*-test). Statistical significance of vasopressin-induced cAMP production in vector-transfected cells was determined with two-way ANOVA with a Dunnett’s multiple comparison test and indicated with #. ^#^*P* < 0.05; ***P < 0.001; ns, not significant.

Activation of V1_a_R by vasopressin leads to classical G_q_ protein recruitment and induction of a downstream intracellular signaling cascade involving phospholipase C activation, IP_3_ production, Ca^2+^ release from intracellular stores and associated PKC activation (Thibonnier et al. 1994). To obtain a direct experimental read-out of V1_a_R activity in mammalian cells, COS-7 cells were transiently transfected with the V1_a_R and V1_a_R-mediated IP accumulation upon exposure to vasopressin and/or hyperosmotic challenges was assessed. Functional expression of the V1_a_R was initially verified by performing a vasopressin dose–response curve of V1_a_R-transfected cells versus vector-transfected control cells. Application of vasopressin had no effect on IP production in vector-transfected control cells but significantly increased the IP accumulation in a dose-dependent manner (EC_50_ = 0.68 nmol/L, 95% CI: 0.44; 1.05, *n* = 7, Fig.3A) to 9.4 ± 1.9-fold in V1_a_R-tranfected cells, significantly higher than that observed in the vector-transfected cells, *n* = 3–8, *P* < 0.001 (Fig.3B). To determine a putative hyperosmolar-induced activation of V1_a_R, vector- or V1_a_R-transfected cells were, in parallel, exposed to a 50 mOsm hyperosmotic challenge either (1) for 30 sec followed by return to control solution, (2) for the entire accumulation period (90 min), or (3) as a gradual 5 mOsm increase every 10 min (Fig.3B). None of these hyperosmolar challenges promoted significant V1_a_R activation as measured by IP accumulation (*n* = 3–8 experiments), indicating a lack of receptor-dependent osmosensing.

It was previously suggested that hyperosmolarity could augment the V1_a_R response to vasopressin (Izumi et al. 2008). To assess a putative synergistic effect of vasopressin and hyperosmolar challenges on the V1_a_R, dose–response curves for IP production were generated in V1_a_R-transfected cells following vasopressin exposure in isosmolar assay solutions or in solutions containing hyperosmolar loads of 50 or 150 mOsm in the assay solution (Fig.3C). 50 mOsm hyperosmotic assay solutions lowered the E_max_ (measured at 1 *μ*mol/L AVP) to 93 ± 2% (*n* = 4, *P* < 0.05), whereas addition of 150 mOsm lowered E_max_ to 76 ± 5% (*n* = 3, *P* < 0.001) compared to isotonic vasopressin exposure (assessed with one-way analysis of variance [ANOVA] with Dunnett’s multiple comparison post hoc test). Therefore, rather than a hyperosmolar-induced *increase* in V1_a_R activity, the combined effect of vasopressin exposure in a hyperosmolar setting resulted in *decreased* V1_a_R activity. Hyperosmotic challenges did, in addition, significantly increase the potency of vasopressin on the V1_a_R: compare EC_50_ of 0.31 nmol/L, 95% CI: 0.22; 0.43, *n* = 4 obtained in the presence of 50 mOsm hyperosmolar challenge with the EC_50_ of 0.68 nmol/L 95% CI: 0.44; 1.05, *n* = 7 obtained in isosmotic control solution, *P* < 0.05.

A range of G protein-coupled receptors display biased signaling, that is, they are capable of signaling through various G proteins and/or other intracellular signaling cascades depending on the specific agonist (Violin and Lefkowitz 2007; Steen et al. 2014). To determine if the V1_a_R, upon a hyperosmolar stimulus, was capable of signaling through an alternative canonical signaling pathway involving G_s_ recruitment, activation of adenylate cyclase and increased cAMP production, vector- and V1_a_R-transfected cells were exposed to vasopressin or to hyperosmolar challenge and intracellular cAMP levels measured. The V1_a_R did not promote cAMP production under any of the experimental conditions (*n* = 4–6 experiments, Fig.3D), suggesting that the V1_a_R is unable to couple to G_s_ following vasopressin exposure or hyperosmolar challenges. We did, however, note a slight vasopressin-induced increase in cAMP production in vector-transfected cells (*n* = 5, *P* < 0.05), indicating the presence of endogenous V2R in the COS-7 cells. This endogenous cAMP production was, however, not sensitive to hyperosmolarity either and did therefore not interfere with our interpretation. These results indicate that the V1_a_R is unable to couple to G_s_ under the present experimental conditions and that it reacts solely to vasopressin exposure, resulting in G_q_ activation, in a manner only slightly affected by simultaneous hyperosmolar challenges in the physiological range.

### Putative osmosensing by V2R assessed by quantification of intracellular secondary messengers

**Figure 4:**
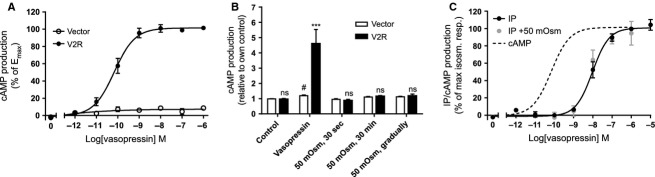
Hyperosmolar effects on V2R-dependent cAMP and inositol phosphate (IP) production. (A) Vasopressin dose–response curve measured with cAMP response of COS-7 cells transfected with either vector alone or with V2R, *n* = 5. (B) cAMP production of COS-7 cells transfected with either vector alone or with V2R upon addition of vasopressin (1 *μ*mol/L) or various hyperosmolar treatments as indicated on graph, *n* = 4–6. (C) IP production (black and gray circles) and cAMP accumulation (dashed line, adapted from panel A) of COS-7 cells transfected with V2R upon vasopressin exposure, *n* = 4–7. Data obtained with V2R-transfected cells were compared to those obtained with vector-transfected cells using two-way ANOVA with Šídák’s multiple comparison post hoc test and indicated with *. Statistical significance of vasopressin-induced cAMP production in vector-transfected cells was determined with two-way ANOVA with a Dunnett’s multiple comparison test and indicated with #. ^#^*P* < 0.05; ***P < 0.001; ns, not significant.

The V2R is related to the V1_a_R, but is abundantly expressed in the kidney where it signals predominantly via activation of G_s_, resulting in increased adenylate cyclase activity and increased cAMP production (Juul et al. 2014). Due to its expression in the kidney collecting duct, the V2R faces a progressively increasing hyperosmotic environment in the transition from cortical to medullary segments, suggesting that hyperosmolarity may regulate V2R function. Thus, in a separate experimental series, the response of the V2R to direct hyperosmotic challenge was determined. COS-7 cells were transiently transfected with the V2R or empty vector and vasopressin-dependent cAMP production was determined. A slight cAMP production was observed in vector-transfected cells upon addition of vasopressin to the assay solution (*P* < 0.05, *n* = 5), although in V2R-transfected cells, the cAMP production increased in a dose-dependent manner (Fig.4A) to 4.6 ± 0.9-fold, which is significantly higher than that observed in the vector-transfected cells, *n* = 5, *P* < 0.001, Fig.4B. The V2R displayed higher potency toward vasopressin (EC_50_ = 0.068 nmol/L, 95% CI: 0.044; 0.107, *n* = 5) than the V1_a_R, *P* < 0.001. To determine a putative hyperosmolar-induced activation of the V2R, vector- or V2R-transfected cells were, in parallel, exposed to a 50 mOsm hyperosmotic challenge either (1) for 30 sec followed by return to control solution, (2) for the entire accumulation period (30 min), or (3) as a gradual 10 mOsm increase every 6 min (Fig.4B). None of these hyperosmolar challenges promoted V2R activation as measured by cAMP production (*n* = 4–6 experiments), indicating lack of receptor-dependent osmosensing.

To determine whether the V2R was capable of signaling through the alternative G_q_ signaling pathway (i.e., the main signaling pathway for the V1_a_R), IP accumulation was determined in COS-7 cells transiently expressing the V2R. At vasopressin concentrations above 1 nmol/L, IP production of the V2R-expressing COS-cells was increased (black symbols in Fig.4C, included for comparison is a dashed line for cAMP production from Fig.4A). The maximal IP production obtained with the V2R-expressing COS-7 cells amounted to 33 ± 3% of that obtained with V1_a_R-expressing COS-7 cells, *n* = 3, *P* < 0.01 (assessed with Student’s *t*-test) and the potency toward vasopressin was significantly reduced (compare EC_50_ = 9.5 nmol/L, 95% CI: 6.5; 13.7, *n* = 7 for IP production with EC_50_ = 0.068 nmol/L, 95% CI: 0.044; 0.107 for cAMP production, *n* = 5, *P* < 0.001). Simultaneous hyperosmolar challenge of 50 mOsm did not affect the IP production of the vasopressin dose–response relationship (gray symbols in Fig.4C): compare EC_50_ of 5.5 nmol/L, 95% CI: 3.8; 8.1, *n* = 4 obtained in the presence of 50 mOsm hyperosmolar challenge with the EC_50_ of 9.5 nmol/L, 95% CI: 6.5; 13.7, *n* = 7 obtained in isosmotic control solution, *P* = 0.20.

## Discussion

An ability of specific vasopressin receptors to respond to local hyperosmotic stress, rather than exclusively being activated by increases in systemically circulating vasopressin would theoretically placate the delay inherent in hyperosmotic-induced vasopressin release. Such “osmosensors” have been detected both peripherally and centrally, mainly in the organum vasculosum of lamina terminalis (OVLT) (Ciura and Bourque 2006; Bourque 2008). The neurons of OVLT respond readily to dehydration through cellular shrinkage and mechanically induced increase in channel opening of the transient receptor potential vanilloid 1 (TRPV1) channel, which via neuronal depolarization leads to systemic and/or central release of vasopressin (Ciura and Bourque 2006; Ciura et al. 2011). Since a prominent role of the vasopressin receptors is to orchestrate cellular and systemic volume regulation, it would be of physiological advantage for the receptors to, in addition, sense the osmotic change directly in the affected cells and initiate the cellular response on the onset of systemic dehydration. In fact, a previous study of V1_a_R-expressing cells reported a marginal release of intracellular Ca^2+^ in response to a hyperosmotic challenge in the absence of vasopressin and a substantial increase in vasopressin-induced intracellular Ca^2+^ release upon vasopressin exposure during a hyperosmotic challenge (Izumi et al. 2008). Here we have resolved whether vasopressin receptors have the ability to respond directly to hyperosmotic challenge by examining V1_a_R-dependent effects upon heterologous expression in *Xenopus* oocytes or in mammalian cells. These cell types were chosen to obtain a cellular system in which the activity of the vasopressin receptors could be determined in isolation with limited contribution of other membrane channels, transporters, or receptors which could putatively display unidentified osmosensing abilities. We aimed to obtain cell shrinkage at a physiological relevant, yet experimentally detectable, level, given the relatively limited osmotic water permeability of *Xenopus* oocytes and COS7 cells, compared to that of the osmosensing brain areas. We therefore opted for a hyperosmolar challenge of 50 mOsm which we demonstrated to provide around 1% cell shrinkage of the oocytes and predicted to provide a maximum of 15% shrinkage of the COS7 cells. By examining V1_a_R-dependent effects on AQP4 function or by quantifying V1_a_R activity both directly (IP production) and indirectly (downstream protein regulation), we observed an absolute requirement for vasopressin to activate the V1_a_R. However, neither osmotic induction of receptor activity nor an osmotic augmentation of the vasopressin-dependent response (at any vasopressin concentration) was observed, although the potency toward vasopressin was slightly increased during an osmotic challenge. These findings contrast those of Izumi and colleagues who employed intracellular Ca^2+^ measurements as their functional read-out of a putative synergistic effect of vasopressin and hyperosmolarity on V1_a_R (Izumi et al. 2008). Although we have not identified the experimental reason for the discrepancy with this study, it is possible that the excessively large hyperosmolar gradient (Δ 200 mOsm) employed in the previous study promoted cellular signaling which would not be apparent at more physiological levels of cell shrinkage. Alternatively, one may speculate whether the open probability of putative endogenously expressed volume-sensitive Ca^2+^ channels, such as TRP-channels, could be modulated by activation of V1_a_R and thereby indirectly generate the observed synergistic effect of hyperosmolarity and vasopressin on the Ca^2+^ signaling. In favor of alternative direct mechanisms of osmosensing, hyperosmolar perfusion directly increased the water permeability of rat inner medullary collecting duct (Nadler 1990; Kudo et al. 1992) even in the absence of vasopressin (Kudo et al. 1992).

V1_a_R activation classically results in G_q_ protein recruitment, PLC*β* activation, inositol triphosphate (IP_3_) generation, and subsequent release of Ca^2+^ from intracellular stores (Thibonnier et al. 1994). However, V1_a_R-dependent excitation of motoneurons in a Ca^2+^-, PLC*β*-, and PKC-independent manner has been demonstrated (Reymond-Marron et al. 2006) and the observed V1_a_R-dependent cAMP production was proposed to originate from G_s_ activation (Wrobel et al. 2011). However, in the present study neither exposure of the V1_a_R to vasopressin nor hyperosmotic stress resulted in alternative signaling through the adenylate cyclase signaling pathway. Although these data indicate that the V1_a_R cannot signal through G_s_ in our experimental system, we cannot exclude that V1_a_R-dependent activation of phospholipase A_2_ and phospholipase D, or direct signaling via the beta-arrestin pathway may act as alternative signal transduction pathways for the V1_a_R (Briley et al. 1994; Terrillon et al. 2004).

Similar to the V1_a_R, the V2R was dependent on the presence of vasopressin for receptor activation and was insensitive to osmotic challenges in the absence of vasopressin. We confirmed the previously reported ability of the V2R to activate G_q_ (Zhu et al. 1994) although with a lower IP production than that of V1_a_R, which may be due to either lower V2R-mediated coupling efficiency toward G_q_ or to lower expression levels of V2R, both compared to that of V1_a_R. V2R displayed lower potency toward vasopressin when signaling through the G_q_ protein than following its classical interaction with G_s_ (140-fold (this study); 35-fold (Zhu et al. 1994)). The V2R-dependent signaling via G_q_ was, as was the case for G_s_ signaling, unperturbed by a simultaneous hyperosmolar challenge. V2R-mediated G_q_-signaling thus occurs exclusively at excessively high vasopressin concentrations.

In conclusion, the V1_a_R and the V2R respond directly to vasopressin exposure and do not share an ability to act as osmo- or volume sensors when exposed to an osmotic gradient in the absence or presence of vasopressin. At higher vasopressin concentrations, the V2R displayed G_q_-dependent signaling, whereas the V1_a_R failed to support G_s_-dependent signaling at the tested ligand concentrations. Neither the V1_a_R nor V2R induced any signaling in response to cell shrinkage. Taken together, vasopressin receptor-mediated cell volume regulation is not initiated via cell shrinkage but rather taking place exclusively upon systemic and/or central release of vasopressin during dehydration.
